# Extracellular membrane particles *en route* to the nucleus – exploring the VOR complex

**DOI:** 10.1042/BST20253005

**Published:** 2025-04-24

**Authors:** Aurelio Lorico, Mark F. Santos, Jana Karbanová, Denis Corbeil

**Affiliations:** 1 Department of Basic Sciences, College of Osteopathic Medicine, Touro University Nevada, Henderson, NV 89014, U.S.A.; 2 Biotechnology Center (BIOTEC), Center for Molecular and Cellular Bioengineering, Technische Universität Dresden, Dresden, Saxony, Germany; 3 Tissue Engineering Laboratories, Medizinische Fakultät der Technischen Universität Dresden, Dresden, Saxony, Germany

**Keywords:** cancer, extracellular vesicles, HIV-1, nucleoplasmic reticulum, spathasome, VOR complex

## Abstract

Intercellular communication is an essential hallmark of multicellular organisms for their development and adult tissue homeostasis. Over the past two decades, attention has been focused on communication mechanisms based on various membrane structures, as illustrated by the burst of scientific literature in the field of extracellular vesicles (EVs). These lipid bilayer-bound nano- or microparticles, as vehicle-like devices, act as regulators in various biological and physiological processes. When EVs are internalized by recipient cells, their membrane and cytoplasmic cargoes can interfere with cellular activities, affecting pathways that regulate cell proliferation, differentiation, and migration. In cancer, EVs can transfer oncogenic factors, stimulate neo-angiogenesis and immunosuppression, reprogram stromal cells, and confer drug resistance traits, thereby remodeling the surrounding microenvironment. Although the mechanisms underlying EV biogenesis and uptake are now better understood, little is known about the spatiotemporal mechanism(s) of their actions after internalization. In this respect, we have shown that a fraction of endocytosed EVs reaches the nuclear compartment via the VOR (VAP-A-ORP3-Rab7) complex-mediated docking of late endosomes to the outer nuclear membrane in the nucleoplasmic reticulum, positioning and facilitating the transfer of EV cargoes into the nucleoplasm via nuclear pores. Here, we highlight the EV heterogeneity, the cellular pathways governing EV release and uptake by donor and recipient cells, respectively, and focus on a novel intracellular pathway leading to the nuclear transfer of EV cargoes. We will discuss how to intercept it, which could open up new avenues for clinical applications in which EVs and other small extracellular particles (e.g., retroviruses) are implicated.

## Introduction

Extracellular vesicles (EVs) are nano- to micro-sized extracellular particles released by all cells, a cellular process conserved throughout evolution. An important structural aspect of EVs is that they are enclosed by a lipid bilayer membrane, reminiscent of retroviruses, which distinguishes them from other non-vesicular extracellular particles [1]. Over the past two decades, EVs have attracted worldwide interest as mediators of intercellular communication, transferring nucleic acids, proteins, and lipids between neighboring cells or over long distance via body fluid flow. Because of their potential clinical relevance and their involvement in numerous cellular and physiological phenomena, they have been the subject of extensive studies and highlighted in various review articles [2–4]. For the historical background of EVs, readers should consult two excellent publications [5,6]. Guidelines and updated versions have been published on how to handle and study these bioparticles, enabling non-specialists in the field to study them in a standardized way [1,7,8].

Two main functions have been attributed to EVs: a clearance process and mediating intercellular communication. These two activities are not mutually exclusive and can influence both donor and recipient cells. Together, they *“may perform a physiological function*“, as Trams and colleagues hypothesized over 40 years ago [9], such as supporting development, maintaining homeostasis, or promoting tissue transformation.

The release of EVs might be a response to, or might regulate, cellular changes in vesicle-producing cells [10,11]. The clearance process has been demonstrated in seminal publications showing the disposal of transferrin receptor proteins during reticulocyte maturation via the discharge of EVs from the endosomal compartment [12,13] (reviewed in Ref. [14]). A similar process can occur at the plasma membrane, as exemplified by the budding of EVs containing the egg-associated Juno protein, that is, the receptor of the sperm Izumo1, directly from the zygote surface upon fertilization [15]. Similarly, the discharge of EVs containing the cluster of differentiation (CD) 133 (alias prominin-1), a cholesterol-binding membrane glycoprotein largely used as an antigenic stem cell marker, occurs during the differentiation process of neuroepithelial or hematopoietic progenitors as well as in cancer cells, may contribute to a down-regulation of signaling pathways regulating stem/progenitor cell properties, and consequently, promote their differentiation [16,17] (reviewed in Refs [18,19]). These and other examples demonstrate that the removal of obsolete membrane proteins by degradation-independent mechanisms in the lysosomal compartment can contribute to cell/tissue maintenance or be a response to the cellular environment [20]. This last aspect also raised the issue of why other clearance mechanisms exist, and addressing this question could indirectly provide an answer or an alternative role for EVs, notably in intercellular communication among others.

By transporting and transferring biomaterials between cells, EVs can propagate morphogens and exchange nucleic acids such as messenger RNAs (mRNAs) and microRNAs; the latter thought to have an impact on post-transcriptional cytoplasmic events in recipient cells [21–23]. EV cargoes can also reach the nuclear compartment of recipient cells and hence affect transcriptional activities [24–29]. Altogether, EVs are now recognized for their contribution to development, modulation of the immune system, and angiogenesis [30–33]. Their interest for regenerative medicine is therefore growing, as they can potentially be used for therapeutic purposes, either in their native or engineered form, to promote tissue reconstruction [34].

In disease states, the aberrant composition of EVs and their excessive cell release have a negative impact on the surrounding tissues [35]. In cancer, the horizontal transfer of oncoproteins and nucleic acids profoundly alters the primary tumor microenvironment, favoring cancer cell migration and transfer of drug resistance. It also promotes the formation of a pre-metastatic niche by altering or reprogramming healthy stromal cells, which, collectively, would lead to the invasive activity and formation of metastases [36–42]. Furthermore, cancer cell-derived EVs have a ‘positive’ impact on neo-angiogenesis and a ‘negative’ one on immune responses, altogether stimulating cancer growth [43–46]. Thus, finding a way to intercept EV-mediated cell–cell crosstalk could lead to new anti-cancer therapies by inhibiting cancer growth at the primary site and/or blocking the formation of metastases. This topic is further discussed below. Of note, EV cargoes, including oncogenes, in various body fluids can serve as biomarkers to monitor the presence and/or progression of cancer cells [47]. As a non-invasive method, liquid biopsy combined with multi-omics approaches could provide information for the diagnosis and prognosis of solid tumors and thus be useful for the disease management at different stages.

Once secreted, EVs can not only be taken up by other cells that are in contact with a given physiological fluid but also be entrapped in the extracellular matrix (ECM), and matrix-bound nanovesicles or matrix vesicles have now been identified as an integral and functional component of ECM bio-scaffolds [48–50]. Whether they should be considered a subgroup of EVs, as opposed to those associated with a body fluid, is still a matter of debate [51]. However, we could consider them as distinct if they act on the ECM as a functional constituent or on other cells that contact them. Within a tissue, EVs can participate in the diffusion of signaling molecules [52,53] even if the cells from which they originate are no longer present and thus create a path that other cells can use to make their way out of a tissue, a phenomenon that could be relevant during the development or in cancer at the stage of intravasation at the primary tumor site. Strikingly, ECM-associated EVs can guide the directional movement of cells by influencing the stability of their membrane protrusions, a function that does not necessarily require EV internalization [54]. Nonetheless, the release of EVs from cancer cells and their binding to ECM may also facilitate their uptake by non-tumorigenic cells and thus affect them [55].

Overall, the world of EVs offers great potential in many spheres of research, from bioengineering to medical applications, which may explain the growing interest in EVs. Efforts to understand them in more detail have led to the recent discovery of new types of EVs and intracellular pathways regulating their formation in donor cells or transmitting their biological messages to recipient cells notably in their nuclear compartment.

### Various types of EVs

**Figure 1:**
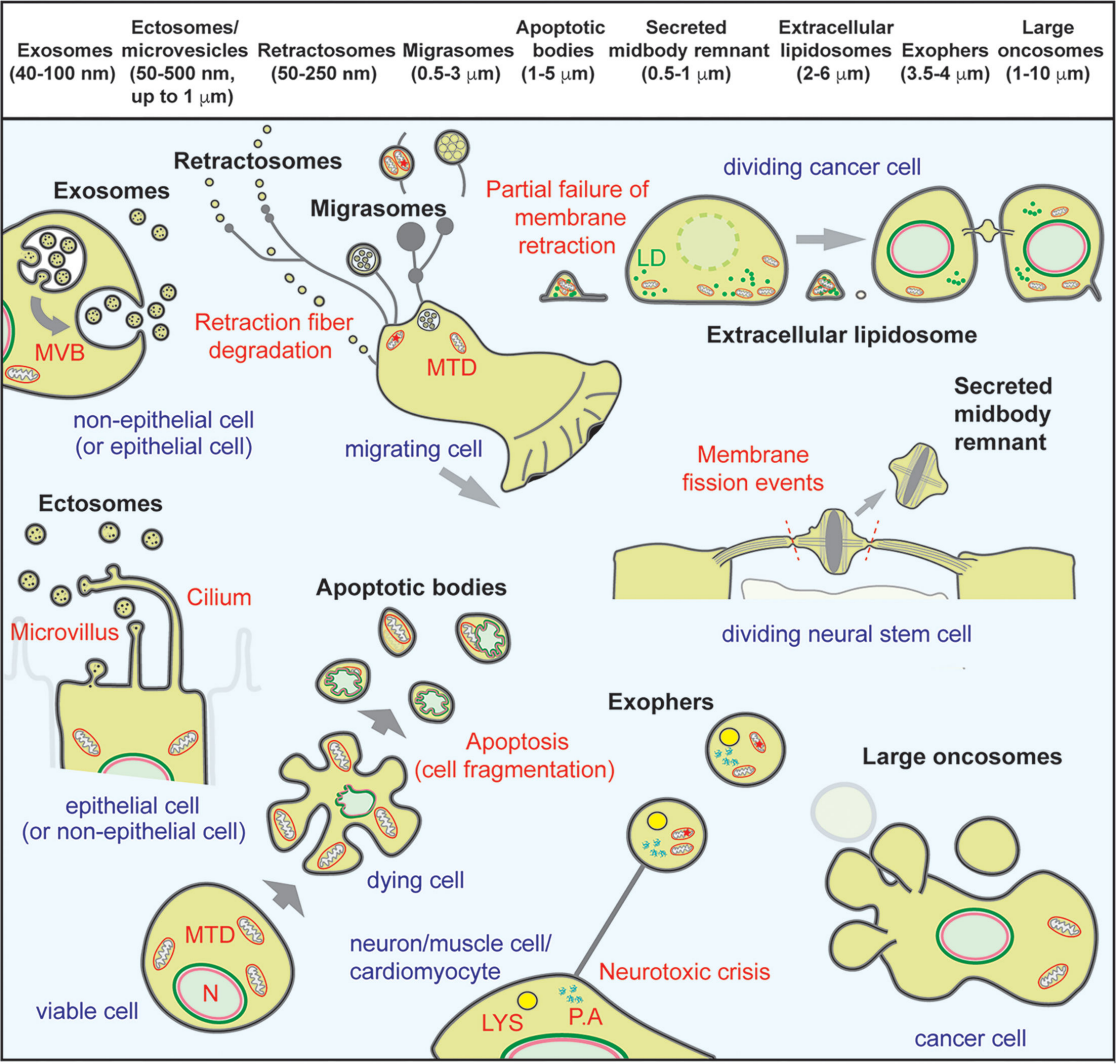
Various types of EVs are generated by distinct cellular mechanisms. EVs are a heterogeneous population of biological particles released from cells, classified according to size (top panel), cellular origin (bottom panel), and composition (not illustrated). The relative diameter of a particular EV type is indicated in parentheses. Small (<150 nm) and large (>150 nm) EVs can coexist in a given body fluid, and a given cell type can release different entities. Small EVs can be discharged as exosomes when MVB containing ILVs fused with the plasma membrane or as ectosomes when they shed directly from the plasma membrane, notably from membrane protrusions such as microvilli and primary cilium. Small EVs, called retractosomes, can also be produced by the degradation of retraction fibers left behind migrating cells. The latter structure can also release larger ectosomes, referred to as (free) migrasomes, which contain numerous small vesicles and/or damaged mitochondria (MTD, red star) or MVBs. Exophers, another large ectosome type, released by neurons and other cells under conditions of stress, also contain damaged mitochondria, lysosomes (LYS) and protein aggregates (P.A). Dividing cancer or stem cells also release large ectosomes, such as extracellular lipidosomes containing mitochondria and lipid droplets (LD), and secreted midbody remnant. The former result from incomplete cell retraction at the onset of mitosis, while the latter arise from a double membrane fission of the central part of the midbody at the end of cytokinesis. Bulky ectosomes known as apoptotic bodies discharged by dying cells in the last stage of apoptosis, and large oncosomes shed from the membrane blebs of cancer cells harboring an amoeboid migration, add to the large spectrum of EVs present in the extracellular milieu. Illustrations are based on results presented in Refs [12,16,53,57–65], and adapted from Ref [66]. EVs, extracellular vesicles; N, nucleus.

The term ‘exosomes’ is probably the most common in the field of EVs, even though they represent only one type of small EVs with a diameter less than 100 (or 120–150) nm. They originate in the endosomal compartment, where inward budding of the limiting membrane into the lumen of the late endosome/multivesicular body (MVB) forms small intralumenal vesicles (ILVs). Upon fusion with the plasma membrane, MVBs release in an exocytic manner into the extracellular space, their ILVs, which are then referred to as exosomes [13] (reviewed in Refs [6,56]) (Figure 1). Recent observations in cancer cells suggest that clusters of exosomes can be released *en bloc* from an MVB-like structure emerging from cellular processes [67–69]. In addition to exosomes, other types of EVs have been described that originate from the plasma membrane and are commonly referred to as ectosomes or microvesicles [70]. They come in different sizes, as small entities, similar to exosomes, and large entities. Small ectosomes, which are difficult to physically separate from exosomes, bud directly from the plasma membrane [71] or from its protrusions, notably microvilli and ciliary structures [16,20,57,72–74] (Figure 1). Another type of small ectosomes, called retractosomes, is produced by the degradation of retraction fibers and remain partially attached to the ECM [75]. Various types of large ectosomes up to 10 μm in diameter have been described and, depending on their origin, mode of formation, specific composition, and/or donor cell status, they have been classified as migrasomes [58], apoptotic bodies [59], secreted midbody remnants [16,57,76], extracellular lipidosomes [60], exophers [61,77], and large oncosomes [62] (Figure 1). The legend to Figure 1 provides further details on these ectosomes, including the cells or their status from which they originate.

The intralumenal cargoes of these distinct EVs vary considerably, particularly in regard to proteins and nucleic acids. Thus, migrasomes that grow along retraction fibers contain and release a chemoattractant (i.e., the C-X-C motif chemokine ligand 12); together with retractosomes, they participate in the formation of particular paths within the ECM [52,53]. Large ectosomes may also contain organelles such as mitochondria, endosomes, and lipid droplets [53,60,61,65,78] (Figure 1). Their release can have an impact not only on donor but also on recipient cells. This is especially true for the extracellular lipidosomes that contain both mitochondria and lipid droplets [60,78], as these two entities can act in synergy, the former being the energy factory and the latter being the energy reservoir [79]. In cancer, accumulation of lipid droplets has been associated with aggressiveness and chemotherapy resistance [80]. Further studies are needed to fully understand the impact of EV-mediated intercellular organelle transfer, along with the precise role(s) of these different types of EVs under normal and pathological conditions.

### EV surface composition and cellular environment dictate EV uptake

The composition of EVs is important, not only to promote or dictate an effect on the recipient cells via bioactive components, but also to favor their interaction with them, and perhaps regulate their internalization. The latter can occur using various mechanisms. Surprisingly, the knowledge that could predict the recipient cells for a given EV is limited. This situation reflects the heterogeneity of EVs in terms of surface composition among others [81]. This is obvious if we consider the different modes of formation of small and large EVs (see above) and the distinct mechanisms that regulate the biogenesis of particular small EVs such as exosomes (or ectosomes) (reviewed in Refs [4,82,83]). Furthermore, specific proteins may participate in independent mechanisms regulating the formation of both types of small EVs, that is, exosomes and ectosomes. For example, tumor susceptibility gene 101 (TSG101), which is part of the endosomal sorting complex required for transport and commonly used to identify exosomes [84,85], is also participating in the arrestin domain-containing protein 1-mediated budding and release of small ectosomes from plasma membrane [86]. Similarly, common markers (e.g., tetraspanins CD9, CD63, and CD81 or pentaspanins CD133 and prominin-2) can be found at various expression levels on the surface of exosomes and ectosomes and influence their uptake by recipient cells, which can lead to different responses [81,87,88] (reviewed in Refs [19,89,90]).

Under physiological conditions, different cell types may collectively contribute to the total amount of EVs in a given body fluid, further complicating the analysis of individual EVs with their recipient cells. Furthermore, it remains to be determined whether they act sequentially or synergistically after internalization, making it even more difficult to assess the actions of EVs *in vivo*. It is worth noting that the EV internalization is not synonymous with functional effect, as they can be released again with their cargo intact after being retained or recycled in the endosomal compartment [91].

Knowledge of the composition of the EV surface is, therefore, essential, as a particular protein (or lipid) could contribute to EV binding/targeting to recipient cells, and perhaps to their selective uptake [92,93], as silencing all or particular EV-associated proteins has been demonstrated to interfere with their internalization [94]. For instance, treatment of exosomes with proteinase K has been shown to reduce their uptake by recipient cells [95,96], while silencing CD9 (tetraspanin-29) in EV donor cells prevents the internalization of derived small CD9–EVs by recipient cells [94]. Particular blocking antibodies directed against specific EV proteins can also interfere with their uptake [97], as we showed with a monovalent antibody (antigen-binding fragment) to CD9 [98]. In contrast, divalent anti-CD9 antibody was shown to promote EV internalization, a process dependent on CD9 expression level in recipient cells [94,98] (reviewed in Ref. [99]). Integrins, adhesion proteins, and metalloproteinases associated with EVs could also contribute to their selective binding [100–103]. In cancer, the organotropic features of metastases may be governed by the integrin expression pattern at the exosome surface [104]. Overall, certain EV surface components as well as peculiar glycan profiles can participate (or favor) the EV–cell binding and determine the underlying mechanism of EV internalization [105]. It is not excluded that the EV–cell interaction might promote the formation of membrane microdomains (e.g., tetraspanin-enriched microdomains, lipid rafts) on one or both adjacent membranes and together with associated proteins trigger EV internalization [89,106,107]. Tetraspanin proteins and lipid raft-associated proteins such as CD9 and CD133 could be instrumental in the membrane reorganization processes [90,108].

In the future, the precise identification of the EV surfactome, combined with artificial intelligence that deciphers all the interconnected protein–protein, protein–lipid, and lipid–lipid interactions, could make it possible, from liquid biopsies, to identify not only donor cells but also target cells and to predict the physiological or pathological evolution of a particular condition [109].

EVs can have an impact on cell behavior, notably migration as highlighted above for those entrapped in the ECM, but also when their contents are transferred to recipient cells. Protruding structures such as filopodia or cilia in recipient cells could be the preferred point of contact with EVs [110,111]. After interaction, it was documented that EVs appeared to move along filopodia toward the cell body at a constant speed corresponding to the retrograde flow of F-actin [53,111,112]. The ‘filopodia surfing’ is somewhat similar to the phenomenon observed with some pathogens, including enveloped viruses, which precedes the viral uptake [113]. By analogy with the retrograde transport of epidermal growth factor (EGF) receptors observed after binding to EGF-coated beads and activation of the receptor tyrosine kinase, the mechanism may involve crosstalk between membrane proteins and filopodial actin filaments [114]. In the above cases, extracellular particle uptake occurs at the base of membrane protrusions – an area of active actin remodeling and therefore a specific hotspot for cell entry.

Numerous internalization mechanisms have been proposed to explain EV uptake by recipient cells notably those based on endocytosis [82,115,116]. They can be dependent on clathrin-coated components [117,118], lipid raft [119], and caveolae [120]. Phagocytosis, notably performed by specialized cells such as monocytes and macrophages, contributed as well to EV uptake, leading to their clearance from circulating systems such as bloodstream [23,121]. Interestingly, the presence of CD47 on the EV surface can allow their escape from phagocytosis by exposing a ‘don’t eat me signal’ [122] (reviewed in Ref. [123]). Non-specific internalization of EVs could nonetheless occur, particularly by macropinocytosis, that relies on recipient cell-derived membrane ruffles resulting in the uptake of extracellular fluids into large endocytic vacuoles [117,124,125]. Finally, in addition to the potential fusion of EVs with the membrane of recipient cells, a process that can be pH-dependent [126,127], it cannot be ruled out that, on initial contact with the plasma membrane of recipient cells, EV interaction may trigger a direct cellular response similar to a soluble ligand–recipient interaction (reviewed in Ref. [128]). Here again, membrane microdomains could be involved in such a signalosome platform [129].

Altogether, various distinct mechanisms of the EV uptake have been described, and they are abundantly illustrated in numerous reviews [82,85,115,130,131]. All these internalization processes are not mutually exclusive, although some may predominate over others depending on the nature and perhaps condition of the recipient cells, as well as the size of the EVs to be internalized or engulfed [132]. Importantly, it remains to be established whether a particular EV profile and/or a given internalization mechanism would guide or determine the intracellular pathway of endocytosed EVs to reach their molecular targets in cytoplasm or nucleus of recipient cells. This last aspect is poorly documented.

### Endosomal escape and nuclear transport of biological EV cargoes

An open question of great importance in the field of EVs is how EV cargoes, particularly those carried by small EVs, can reach their molecular targets in the cytoplasm and nuclear compartment of recipient cells without undergoing lysosomal degradation [133,134]. Concentration of EV cargoes at specific cytoplasmic or nuclear targets is essential for a biological effect because the quantity of bioactive materials encapsulated in a single EV is extremely low. An interesting study has revealed that internalization of small EVs is a low-yield process, at least in some cell types, suggesting that the delivery of their cargo must be highly targeted [135]. The same report also points out that solely 30% of endocytosed EVs release their contents into the host cytoplasm after fusing with the endosomal membrane – a process similar to the back-fusion (i.e., (retro)fusion of an ILV with endosomal limiting membrane) that depends on endosomal acidification [135] (reviewed in Ref. [136]). Neutralization of endosomal pH or accumulation of endosomal cholesterol can lead to a block in the release of the EV cargoes [137]. The low endosomal escape process has also been observed in a more artificial system, where the delivery of encapsulated short interfering RNAs, mediated by small lipid nanoparticles, occurs from endosomes to cytoplasm with an efficiency of only 1–2% and during a limited time window within a specific compartment that shares the characteristics of early and late endosomes [138]. Other scenarios have also been proposed to explain endosomal escape, such as transient pore formation or osmotic swelling of endosomes following membrane lysis, the latter processes being promoted by the modulation of intra-endosomal lumenal milieu (reviewed in Refs [139,140]). These observations imply that the release (or discharge) of EV cargoes is a rate-limiting step and might be regulated at the intracellular level, and that endocytosed EVs might perhaps influence the fate of endosomes despite being located in their lumen. Overall, the correct positioning of EV-laden late endosomes close to molecular targets for the EV cargoes could be essential for triggering a cellular response.

Intracellular transport and positioning of late endosomes *en route* for their fusion with lysosomes were demonstrated to involve various cytoplasmic protein partners, whose interactions are regulated by the endosomal cholesterol level. Among them, the small GTPase Rab7, Rab7 effector Rab7-interacting lysosomal protein (RILP), and cholesterol sensor protein – oxysterol-binding protein (OSBP)-related protein 1L (ORP1L) – form a multiprotein complex with the p150^Glued^ subunit of the dynein–dynactin motor complex and regulate microtubule (MT) minus-end transport of late endosomes under high-cholesterol level [141–144]; under low-cholesterol conditions, the OSBP-related domain (ORD) of ORP1L is released from endosomal membranes and reveals its two phenylalanines in an acidic tract (FFAT) motif for the binding of the endoplasmic reticulum (ER) vesicle-associated membrane protein-associated protein A (VAP-A), a molecule involved in the tethering of the ER membrane with numerous organelles including late endosomes (reviewed in Refs [145–147]). This interaction terminates the interaction between p150^Glued^ and RILP. Thus, the level of cholesterol in late endosomes dictates the conformation of ORP1L and subsequently the VAP-A interaction and ER-late endosome contact sites [143]. As a result, the intracellular transport of EV-laden late endosomes involves a stop-and-go movement along the ER, before encountering lysosomes [111].

Of note, VAP-A and ceramide transfer protein CERT, present at the contact sites between the ER and late endosomes/MVBs, promote the incorporation of specific RNAs (e.g., microRNAs let-7a and miR-100) into the nascent ILV and thus participate in the selective loading of exosome precursors [148]. By inducing membrane curvature, ceramide is known to be involved in the biogenesis of ILVs [149]. Similar phenomena can occur with other VAP-A interactors, lipid transporters such as ORP1L that regulate ILV formation and their content [150–152] (reviewed in Ref. [153]). In addition to nucleic acids, ILV sorting of certain cargo proteins such as RNA-binding proteins and the cholesterol-binding membrane protein CD133 (see above) are negatively affected by the absence of VAP-A [154]. As CD133 can interact with TSG101 and syntenin-1 [155,156], the latter associated with syndecan and ALG-2-interacting protein X [157], a large condensate formed by numerous protein–protein/lipid interactions can occur in the contact zone between ER and the late endosome. Such a spatiotemporal complex may regulate the formation and composition of ILVs and perhaps influence their subsequent fate as either lysosomal degradation or release as exosomes under certain cellular conditions.

**Figure 2:**
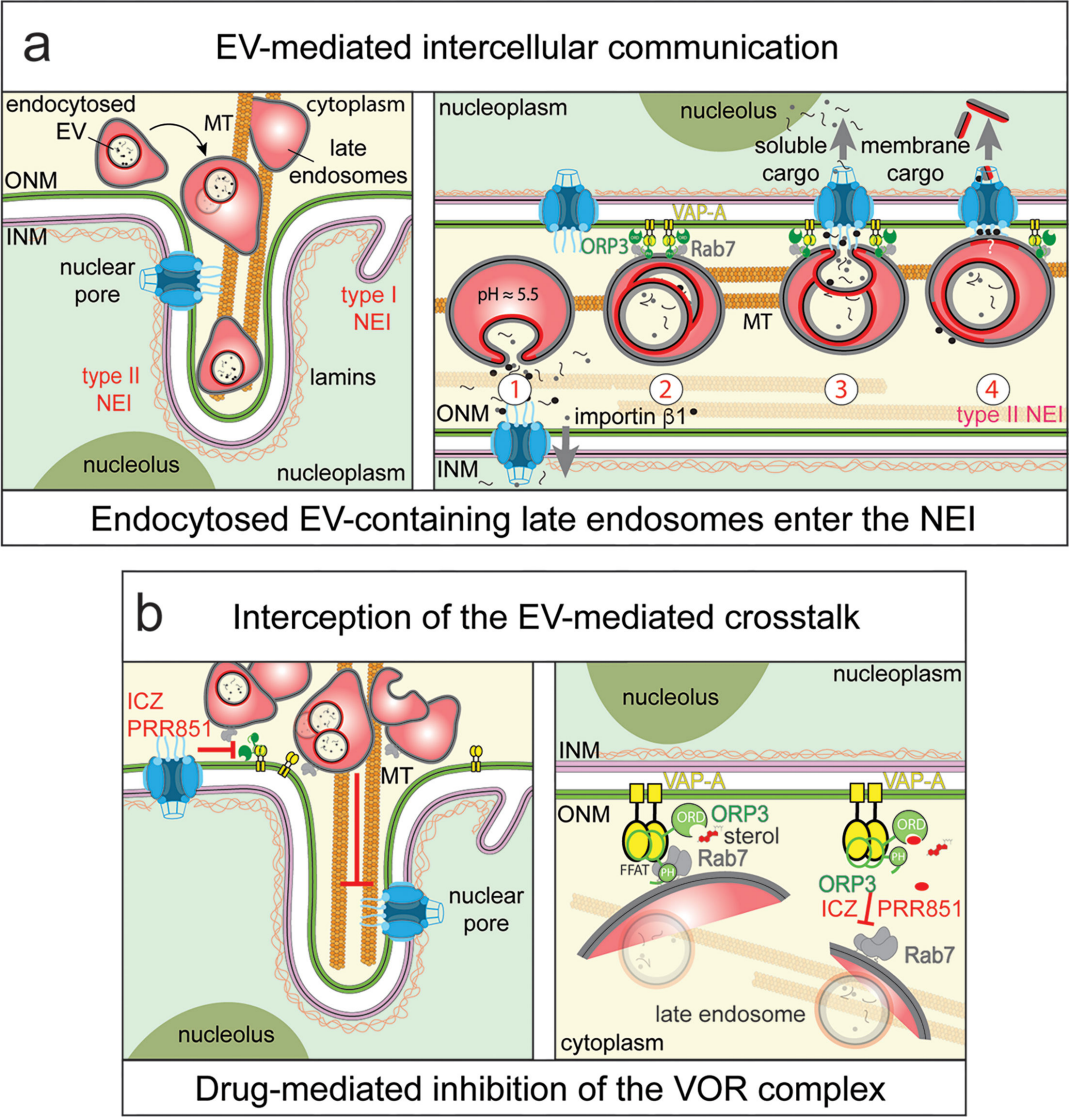
Endocytosis of EVs and their transport to the nucleoplasmic reticulum. (**a**) After endocytosis by recipient cells, small EVs successively reach early and Rab7^+^ late endosomes, and a fraction of the latter enters the nucleoplasmic reticulum via the type II nuclear envelope invagination (NEI, left panel). This transport is MT-dependent and requires the action of a protein complex (called the VOR complex) formed by the ONM-associated membrane protein VAP-A, the phosphorylated form of the cytoplasmic protein ORP3 and Rab7 (right panel). Within NEI, EV cargoes can be released into the cytoplasm upon the acidic pH-mediated fusion of endocytosed EV with late endosomal membrane (#1). There, EV cargoes can then be transferred to the nucleoplasm through the nuclear pore, where they can regulate or influence gene expression. Importin β1, especially those associated with EVs, may play a role in the nuclear import. Alternatively, docking of late endosomes to the ONM could facilitate the EV cargo release (#2). It remains to be determined whether a protein of the VOR complex interacts with components of the nuclear pore, which could facilitate and/or direct the nuclear transfer of soluble EV cargoes (#3). The mechanism regulating the transfer of membrane cargoes from EVs into the nucleoplasm remains to be elucidated, in particular their extraction from the late endosome/EV membrane (#4, question mark). (**b**) Translocation of late endosomes containing endocytosed EVs into a NEI can be intercepted by chemical drugs such as itraconazole (ICZ) or PRR851 (left panel). These drugs interact with the ORD of ORP3, preventing the interaction of the VAP-A-ORP3 complex with Rab7 and hence the docking of late endosomes to the ONM (right panel). Illustrations are based on results presented in Refs [94,161–163]. EVs, extracellular vesicles.

In addition to the cytoplasmic localization of small EV-laden late endosomes, we have previously observed that a minute fraction of them translocate into the nucleoplasmic reticulum, often reaching a deep nuclear localization and terminating near the nucleolus [94]. More specifically, Rab7^+^ late endosomes enter type II nuclear envelope invaginations (NEIs), formed by the inner (INM) and outer (ONM) nuclear membranes [158–160] (Figure 2a, left panel). Type I NEIs are generated solely by INM, and both classes of NEI can co-exist in a given nucleus. NEIs are found in cultured cells or in tissues [94,158,164], and their number increases in cancer, and surprisingly, upon exposure to EVs, suggesting that the latter contributed to their formation (see below) [94,165,166]. Cytoskeletal elements including actin, tubulin, and intermediate filaments are described at the cytoplasmic side of type II NEIs [158,167,168], while a stable lamina is found on the nucleoplasmic side [169]. Nuclear pores are found in type II NEIs, suggesting their potential role in the nucleo-cytoplasmic transport [94,170]. Calcium signaling and nuclear lipid metabolism have been associated with the nucleoplasmic reticulum [158,171]. The presence of ribosomes and ER components in the cytoplasmic core suggests that mRNA translation may take place there [172]. In contrast to Rab7, small GTPase Rab5 and lysosomal-associated membrane protein 1, markers of early endosomes and lysosomes, were excluded from the NEIs [94,161], suggesting that the confinement of Rab7^+^ endosomal structures, albeit a fraction, within the NEI may protect them from fusion with lysosomes and thus from degradation. It will be interesting to further characterize the late endosomes associated with NEI, as they could represent a subpopulation of late endosomes at distinct stages of maturation [173–175].

The relationship between endosomes and the nuclear compartment has also been reported in other systems, where early (Rab5^+^) endosomes in close contact with the nuclear envelope or within the NEI have been observed [176,177]. For instance, following the binding to the cell surface low-density lipoprotein receptor-related protein 1 and endocytosis, the Pseudomonas exotoxin A, a bacterial protein, can reach the nuclear compartment through the docking and fusion of early endosomes with the ONM [176]. Such non-degradative pathway has also recently been illustrated in neurodegenerative diseases such as polyglutamine (polyQ), where early endosomes containing aberrant proteins with a long polyglutamine tract enter the NEIs and deliver pathological polyQ aggregates into the nucleoplasm [177]. For further details on the role of early endosomes in the nuclear trafficking of cargo proteins, please see the following review article [178].

Biologically, the fusion of the endocytosed EV with the limiting membrane of late endosomes located inside a NEI will release EV cargoes into the cytoplasmic core of this narrow compartment, facilitating either their shuttle to the nucleoplasm through the nuclear pore, where they can affect transcriptional activities as demonstrated with cancer cell-derived EV-primed mesenchymal stromal cells [94], and/or their encounter with their cytoplasmic targets, such as mRNAs in the case of EV-derived microRNAs, or the translation machinery in the case of EV-derived mRNAs (Figure 2a, right panel, #1). The role of nuclear pores was revealed by their inhibition with a drug, resulting in the absence of nuclear transfer of EV proteins [94]. For more details, see legend of Figure 2.

Because of the appearance of late endosomes penetrating the nucleoplasmic reticulum, which, when stained with Rab7 and the INM-associated SUN-domain containing protein 2 marker, is morphologically reminiscent of a sword in its scabbard, these double-organelle structures have been called ‘Spathasomes’ (from the Greek and Latin words ‘spathi/spatha’ for sword) [94].

### The VOR complex and its inhibition

In our search for the proteins involved in the docking and dynamic entry of Rab7^+^ late endosomes into the nucleoplasmic reticulum, we discovered that VAP-A and Rab7 are involved in this process, which depends on an intact MT network [162], in some ways similar to the event (ER-endosome interaction) that occurs in the cytoplasmic compartment (see above) [143]. Thus, it appears that VAP-A is involved in both cargo loading into nascent ILVs of donor cells and their discharge from endocytosed EVs associated with late endosomes into NEIs of recipient cells. Although further studies are needed to dissect all facets of VAP-A in these issues, the latter aspect is consistent with the continuity of the ER membrane with the ONM. However, ORP1L is absent in NEI, while the cytosolic OSBP-related protein-3 (ORP3), another member of the OSBP family [179], is present and binds to VAP-A but not to its homologue VAP-B [162]. The VAP-A–ORP3 complex interacts with Rab7 associated with the late endosomes, creating a tripartite complex called VOR (an acronym for the three proteins involved) [162]. Phosphorylation of ORP3 is essential for its binding to VAP-A [180] and consequently to Rab7 [163]. These three proteins co-localize in discrete regions in the nucleoplasmic reticulum, suggesting the potential docking of late endosomes with ONM [162] (Figure 2a, right panel, #2). Yet, it remains to be determined whether the nuclear pores (or certain nucleoporins) participate with the VOR complex in the ONM docking of late endosomes, which could enable correct positioning and facilitate nuclear transfer of the soluble EV cargo after the fusion of endocytosed EVs with limiting membrane of late endosomes (Figure 2a, right panel, #3). As shown for the EV-associated CD9 protein, the extraction mechanism of EV membrane cargo from the endosomal membrane prior to nuclear transfer via nuclear pores remains to be determined (Figure 2a, right panel, #4), as well as the question whether endosomal membrane microdomains are involved [181]. Membrane cholesterol associated with late endosomes and ORP3 may play a role in these processes, as ORP3 binds to cholesterol and 25-hydroxycholesterol in living cells possibly via its ORD, the latter harbors a β-barrel-like fold containing a hydrophobic pocket that might accommodate a sterol molecule [182]. The binding of ORP3 to sterols has been demonstrated using radioactive photoactivatable analogues [182]. Interestingly, only the full-length ORP3, and not its truncated form containing solely the ORD, binds to sterols [182], suggesting that the lack of membrane interaction of the truncated ORP3 ORD mutant has an impact on its association with cholesterol/25-hydroxycholesterol or that a conformational change occurs in the full-length version to allow the binding to sterols. In another context, ORP3 was shown to mediate the trafficking of ciliary cholesterol from peroxisomes to primary cilia [183]. These experimental observations contrast with a previous molecular modeling study [184] and, more recently, the crystal structure of ORP3 ORD, which suggests that its hydrophobic pocket is incompatible with tetracyclic rings, even though its capacity is 2.6 times greater than that of the cholesterol molecule [185,186]. Whether post-translational modifications of ORP3, for example, its phosphorylation or its interaction with VAP-A and/or endosomal membrane, can alter its conformation with respect to its ORD remains to be determined. Although inhibitory competition between 25-hydroxycholesterol and ORD-modeled drugs has been described (see below), the possibility that sterol binding occurs at another ORP3 site cannot be completely excluded.

As EVs are of interest in the field of intercellular communication and could have an impact on various diseases, and in particular in cancers [187], where cells release a greater amount of EVs and promote the transformation of tumor microenvironments, intercepting their internalization by recipient cells and/or their intracellular pathways *en route* to their targets could offer alternatives to block their action [163,188]. In such context, we found that the VOR complex formation can be impeded by the FDA-approved antifungal azole itraconazole (ICZ) through its interaction with the ORD of ORP3 [163] (Figure 2b), which is in line with its action on other OSBP proteins [189]. More precisely, ICZs interfere with the interaction of the VAP-A-ORP3 complex with Rab7, leading to the absence of late endosomes in the nucleoplasmic reticulum (Figure 2b) and, consequently, the nuclear transfer of EV cargoes and the resulting transformation of cancer cells *in vitro* [163].

Molecular modeling of the ORP3 sterol pocket with ICZ has enabled the design of smaller triazole-derivative compounds (e.g., PPR851) lacking the reactive dioxolane moiety responsible for antifungal activity but retaining the effects of ICZ in intercepting the nuclear transfer of EV cargoes [163,190]. Furthermore, the activities of ICZ and PPR851 in inhibiting VOR complex formation were blocked by 25-hydroxycholesterol in a concentration-dependent manner, implicating ORP3 ORD in this process [163]. Homology modeling analyses revealed that the key residues (Arg558, Tyr593 and Trp653) of ORP3 are present and/or in a configuration accessible for drug binding only in ORP7, being absent in OSBP, ORP1, ORP2, ORP4, ORP6, ORP9, thus limiting potential toxicity. Preliminary data indicate that VOR complex inhibitors have no significant effect on cell growth *in vitro* or on biochemical abnormalities *in vivo* following injection in mice [163,190]. These new chemical compounds could open up a new avenue for limiting the negative effects of EVs in diseases, and further *in vivo* research with animal models is the next step in conceptualizing such an approach.

Altogether, after internalization into recipient cells, endocytosed small EVs, particularly their cargoes, can be delivered to molecular targets located either in the cytoplasm or nucleoplasm, and the latter can be regulated by a new intracellular pathway (Spathasomes) involving the tripartite VOR complex. Its inhibition can hinder EV-mediated intercellular communication and, consequently, their negative impact on the cellular transformation, for example, in the cancer microenvironment [163]. It has yet to be assessed whether this novel pathway is also used during development, where both small and large EVs may play a role [16,29,57,191,192]. To our knowledge, the question of whether cargoes of large EVs, once internalized, reach the nuclear compartment has not been fully addressed.

### The VOR complex beyond EVs: a viral vision

Beyond the field of EVs, the Spathasome and the associated VOR complex could be of particular interest where other types of extracellular particles are engaged. This led us to wonder whether retroviral particles (i.e. enveloped RNA viruses) that are comparable in size and density to small EVs, could shuttle into the nuclear compartment of infected cells using the newly described pathway to deliver their genome, gain access to the host cell’s DNA machinery and begin replication. To this end, we investigated whether human immunodeficiency virus 1 (HIV-1) exploits or hijacks the Spathasome pathway to ensure its complex life cycle, leading to a productive infection. While early reports supported pH-dependent cellular entry of HIV-1 by direct fusion with the plasma membrane, later evidence implies viral fusion with the endosomal membrane after their internalization [193,194]. Using native or CD4-transfected HeLa cells and phytohaemagglutinin/interleukin-2–activated CD4^+^ T cells as host cells and HIV-1 pseudotyped with vesicular stomatitis virus G or native Env protein, we discovered that HIV-1 reached NEIs by a process dependent on the integrity of the VOR complex, as silencing VAP-A and ORP3 prevented viral particle transport in this subnuclear compartment and the presence of its integrase in the nucleoplasm, resulting in the inhibition of productive infection [161]. The role of the VOR complex in this process was confirmed by the application of ICZ or PRR851 [161]. The latter may represent a prototype of a novel class of HIV-1 drugs for the prevention of acquired immune deficiency syndrome.

The use of CD4^+^ T cells was also instructive in relation to the cellular and molecular events involved in this new pathway. It turns out that T cell activation and HIV-1 infection are necessary for the formation of NEIs, as these nuclear membrane subdomains are absent in quiescent T cells, in the absence or presence of HIV-1 infection, as well as in uninfected activated CD4^+^ T cells [161]. Thus, virus-laden Rab7^+^ late endosomes promote the formation of type II NEIs in activated T cells, a phenomenon that parallels the increase in the number of NEIs in cells exposed to EVs (see above). These observations indirectly suggest that the loading of the endosomal system with extracellular particles (EVs or retroviruses) may represent a novel ‘pushing in’ mechanism underlying the biogenesis of NEIs [195]. To establish whether a certain threshold of endocytosed extracellular particles or a specific pathway of internalization as discussed above are required to induce such a process, a more detailed analysis is needed. At the molecular level, ORP3 has been found to be instrumental in this process, as T cell activation stimulates its phosphorylation via protein kinase C, leading to the formation of the VOR complex during HIV-1 infection [161]. This new pathway can therefore be controlled, and it has been proposed that the (hyper)phosphorylation of ORP3 induces a conformational change leading to the exposure of its pleckstrin homology (PH) domain and FFAT motifs [196], which could mediate its dual interactions with the membrane of late endosomes and VAP-A at the ONM [161,163] (Figure 2b). The PH domain has a high affinity to phosphoinositides, which are involved in numerous cellular functions notably membrane dynamics, intracellular trafficking, and signaling among others [197].

The release of viral components from late endosomes into the cytoplasmic core of induced NEIs close to the nuclear pore would therefore position their transfer to the nucleoplasm in a similar way as described above for the EVs. It remains to be determined whether the extreme curvature of the NEIs would facilitate the passage of an intact 60-nm viral capsid through the nuclear pores [198,199], where the diameter of the central channel has been proposed to have a diameter of about 40 nm in general [200]. Capsid uncoating at nuclear pores or inside the nucleus were documented [201,202].

On the whole, as HIV-1 takes advantage of NEIs to facilitate delivery of its viral core into the host nucleus, the role of the Spathasome and VOR complex seems to extend beyond the EV field and may regulate the overall nuclear transfer of extracellular particles taken up by recipient/infected cells. These observations added another link between retroviruses and EVs, in line with the Trojan hypothesis, according to which retroviruses use pre-existing non-viral pathways that regulate exosome biogenesis and uptake [203,204].

In conclusion, the recent discovery of Spathasomes and the associated VOR complex opens up a new avenue to explain the delivery of biomaterials embedded in extracellular particles to the nucleus of host cells, which may be important particularly during development and in certain diseases, where the use of EVs or viral particles is amplified. Further studies are needed to understand all the facets of this new cell pathway, as well as its possible role, along with other pathways, in the nuclear shuttling of other cellular components such as plasma membrane proteins (e.g., CD133, growth hormone receptors) [205,206]. The identification of all partners of the VOR complex that can act synergistically could lead to new possibilities for inhibiting its function.

**Perspectives**

- Highlight the importance of the field – Extracellular vesicles (EVs) have attracted worldwide interest as mediators of intercellular communication, bringing hope for tissue engineering and regenerative medicine, as well as for the treatment of various diseases, including cancer. By stimulating EV release or intercepting their activity, it is possible to influence cell fate, including proliferation, differentiation, and migration.
- Summary of the current thinking – In cancer or other diseases that involve extracellular particles such as EVs and enveloped viruses, blocking the nuclear action of their cargoes by inhibiting the VOR complex in recipient/infected cells could offer a novel alternative for attenuating the disease’s progression.
- Comment on future directions – A deeper understanding of the newly discovered Spathasome pathway and the associated VOR complex could lead to new therapeutic approaches, not only for interfering with EV/virus action but also for promoting nuclear targeting of engineered particles (e.g., liposomes) acting as drug vehicles by conveying specific gene regulators or chemical drugs.

## Abbreviations

CD: cluster of differentiation
ECM: extracellular matrix
EGF: epidermal growth factor
ER: endoplasmic reticulum
EV: extracellular vesicle
FFAT: two phenylalanines in an acidic tract
HIV-1: human immunodeficiency virus 1
ICZ: itraconazole
ILVs: intralumenal vesicles
INM: inner nuclear membrane
MT: microtubule
MVB: multivesicular body
NEI: nuclear envelope invagination
ONM: outer nuclear membrane
ORD: OSBP-related domain
ORP3: oxysterol-binding protein-related protein-3
ORP1L: oxysterol-binding protein (OSBP)-related protein 1L
OSBP: oxysterol-binding protein
PH: pleckstrin homology
RILP: Rab-interacting lysosomal protein
TSG101: tumor susceptibility gene 101
VAP-A: vesicle-associated membrane protein-associated protein A
mRNA: messenger RNA
polyQ: polyglutamine
